# Bacillary Angiomatosis

**DOI:** 10.4269/ajtmh.13-0561

**Published:** 2014-09-03

**Authors:** Fernando Mejía, Carlos Seas

**Affiliations:** Instituto de Medicina Tropical Alexander von Humboldt, Universidad Peruana Cayetano Heredia, Lima, Peru; Departamento de Enfermedades Infecciosas Tropicales y Dermatologicas, Hospital Nacional Cayetano Heredia, Lima, Peru

**Figure 1.**
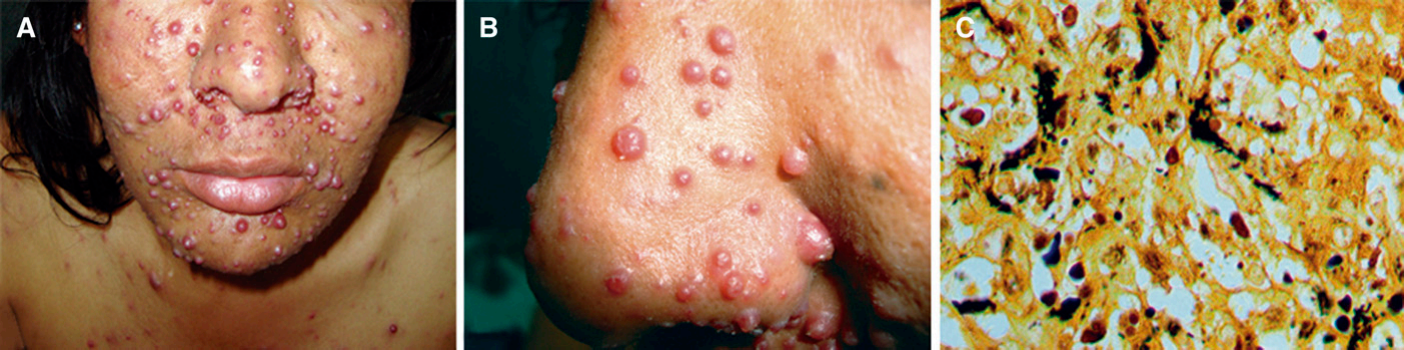
(**A**) Multiple erythematous nodules on the face. (**B**) Close-up view of multiple exophytic nodules. (**C**) Clumps of tangled, dark bacilli on a Warthin–Starry stain (×100).

A 29-year-old man presented with generalized, painless, erythematous lesions and fever. The patient had recently been diagnosed with human immunodeficiency virus (HIV) infection. His CD4 T-cell count at presentation was 14 cells/mm^3^, and he had been started on antiretroviral therapy 15 days before onset of disease. The patient reported illicit drug use and alcohol consumption and had regular contact with cats. On physical examination, the patient was found to have countless scattered, erythematous, violaceous nodular lesions ranging from 5 to 20 mm in diameter (Figure 1A

and B) along with hepatosplenomegaly. A diagnosis of bacillary angiomatosis was made based on skin biopsy histopathology, which revealed clusters of tangled bacilli on a modified silver stain (Figure 1C), and a positive polymerase chain reaction (PCR) for *Bartonella* spp. targeting the 16S-23S ribosomal RNA gene intergenic transcribed spacer. Bacillary angiomatosis is a reactive vasoproliferative lesion that usually occurs in immunocompromised patients in response to infection by *Bartonella* species.^1^ The lesions are clinically indistinguishable from Kaposi's sarcoma and pyogenic granuloma. The clinical presentation after the initiation of antiviral therapy in this patient suggests immune reconstitution inflammatory syndrome. Erythromycin or tetracyclines are the antibiotics of choice.^2^ The patient was treated with doxycycline until the CD4 cell count was above 200 cells/mm^3^, resulting in complete resolution of lesions.
